# Growth factor receptor and β1 integrin signaling differentially regulate basal clonogenicity and radiation survival of fibroblasts via a modulation of cell cycling

**DOI:** 10.1007/s11626-022-00656-z

**Published:** 2022-02-22

**Authors:** Anne Vehlow, Nils Cordes

**Affiliations:** 1 OncoRay – National Center for Radiation Research in Oncology, Faculty of Medicine Carl Gustav Carus, Technische Universität Dresden, Fetscherstr. 74, PF 41, 01307 Dresden, Germany; 2 Department of Radiotherapy and Radiation Oncology, University Hospital Carl Gustav Carus, Technische Universität Dresden, Fetscherstr. 74, PF 50, 01307 Dresden, Germany; 3 Helmholtz-Zentrum Dresden - Rossendorf, Institute of Radiooncology – OncoRay, Bautzner Landstr. 400, 01328 Dresden, Germany; 4 German Cancer Consortium (DKTK), Partner Site Dresden, and German Cancer Research Center (DKFZ), Heidelberg, Germany, Im Neuenheimer Feld 280, 69192 Heidelberg, Germany

**Keywords:** beta1 integrin, Growth factor receptor, Cell cycle, Ionizing radiation

## Abstract

Cell adhesion to extracellular matrix proteins mediates resistance to radio- and chemotherapy by activating integrin signaling. In addition, mutual and cooperative interactions between integrin and growth factor receptor signaling contribute to the cellular radiation response. Here, we investigate to which extend the crosstalk between β1 integrins and growth factor receptor signaling determines the cellular radiation response of fibroblasts by assessing clonogenic survival and cell cycling. By utilizing growth factor signaling competent and either β1 integrin wildtype GD25β1A fibroblasts or β1 integrin mutant, signaling incompetent GD25β1B fibroblasts, we show basal clonogenic survival to depend on growth factor receptor but not integrin signaling. Our data further suggest the cooperation between β1 integrins and growth factor receptors to be critical for enhancing the radiation-induced G2/M cell cycle block leading to improved clonogenic radiation survival. By pharmacological inhibition of EGFR and PI3K, we additionally show that the essential contribution of EGFR signaling to radiogenic G2/M cell cycle arrest depends on the co-activation of the β1 integrin signaling axis, but occurs independent of PI3K. Taken together, elucidation of the signaling circuitry underlying the EGFR/β1 integrin crosstalk may support the development of advanced molecular targeted therapies for radiation oncology.

**Supplementary Information:**

The online version contains supplementary material available at 10.1007/s11626-022-00656-z.

## Introduction

The extracellular matrix (ECM) is an important element of the tumor microenvironment that drives cancer therapy resistance ( Meads *et al*. 2009; Pickup *et al*. 2014; Vehlow *et al*. 2016). Although several therapeutic intervention strategies against cell-ECM-linking molecules have not been successful to date, a large number of preclinical studies show the feasibility and efficiency of such approaches. Others, such as this one, shed more light onto the underlying mechanisms.

By providing anchorage and architectural support, several different cell adhesion molecules connect tumor cells with the ECM. Integrins are the most prominent group of heterodimeric transmembrane adhesion receptors, comprised of 18 alpha (α) and 8 beta (β) subunits making up 24 integrin receptor pairs with specificity for different ECM ligands (Humphries *et al*. 2006). By transmitting mechanical and biochemical cues, integrins regulate survival, migration, and proliferation as well as many other functions in normal and tumor cells (Desgrosellier and Cheresh 2010; Hamidi and Ivaska 2018; Kechagia *et al*. 2019). Integrin-mediated adhesion to the ECM and the associated downstream signaling are a known crucial and per se druggable cause of resistance to genotoxic agents, such as ionizing irradiation and cytotoxic drugs (Damiano *et al*. 2001; Cordes and Meineke 2003; Vehlow *et al*. 2016). Especially, signaling events downstream of β1 integrins involving focal adhesion kinase (FAK), integrin-linked kinase (ILK), and the phosphatidylinolsitol-3-kinase (PI3K)/Akt axis mediate radio- and chemoresistance in many tumor entities (Hehlgans *et al*. 2007; Eke *et al*. 2010; Graham *et al*. 2011).

In addition to providing structural support, the ECM serves as a reservoir for growth factors leading to the activation of pro-survival signaling (Taipale and Keski-Oja 1997; Hynes 2009). Many growth factor receptors, such as the epidermal growth factor receptor (EGFR), are main drivers of resistance to radio- and chemotherapy and potential targets for individualized combination therapies (Bonner *et al*. 2006; Karapetis *et al*. 2008; Zhou *et al*. 2011; Higgins *et al*. 2016). Based on these findings, the cooperative and mutual interactions between EGFR and integrins have been elucidated and how they elicit a therapy-refractory state in cancer cells (; Moro *et al*. 1998; ; Morello *et al*. 2011; Petras *et al*. 2013). For an even better understanding of the exact mechanisms of this receptor crosstalk and its therapeutic exploitability, further studies are desperately warranted.

In this regard, we have documented that β1 integrin and growth factor receptor signaling cooperatively mediate resistance to ionizing irradiation in normal fibroblasts and tumor cells (Cordes *et al*. 2006; Eke *et al*. 2015). Intriguingly, β1 integrin signaling preserved the cellular functionality upon growth factor receptor depletion indicating a compensatory function of integrins in the absence of functional growth factor receptors (Cordes *et al*. 2006). This crosstalk is mainly mediated through PI3K/AKT and MAPK signaling, but seems to involve further yet to be determined signaling networks (Cordes *et al*. 2006; Eke *et al*. 2015). Here, we took advantage of the murine GD25 fibroblast model, of which GD25β1A express signaling competent, wildtype β1 integrin, and GD25β1B express signaling incompetent, mutated β1 integrin. We investigate to which extent the crosstalk between β1 integrins and growth factor receptor signaling determines the cellular radiation response by assessing clonogenic survival and cell cycling as one of the critical processes allowing time for the repair of radiogenic DNA damage. To differentiate between β1 integrin–mediated adhesion and growth factor receptor signaling, fibroblasts were cultured in presence and absence of fibronectin and growth factors. We observed the enhanced radiation-induced G2/M cell cycle arrest to depend on the cooperation of β1 integrins and growth factor receptors. Furthermore, our data suggest that the influence of EGFR signaling on the reported radiogenic G2/M cell cycle arrest co-depends on the activation of the β1 integrin signaling axis. These results underline the importance of untangling cooperative integrin and growth factor receptor networks for further refining their functional consequences on the level of the cellular radiation response and their therapeutic exploitability.

## Materials and methods

### Cell culture

R. Fässler (Max-Planck Institute of Biochemistry, Martinsried, Germany) kindly provided the GD25β1A cells. S. Johansson (Uppsala University, Sweden) generously provided the GD25β1B cells. GD25β1A and GD25β1B were generated from GD25 fibroblasts derived from β1 integrin null stem cells by stably transfecting cDNAs encoding the murine integrin subunit β1A and β1B, respectively (Fassler and Meyer 1995; Wennerberg *et al*. 1996; Armulik *et al*. 2000). Cells were cultured in Dulbecco’s modified Eagle’s medium (DMEM, Thermo Fisher Scientific, Waltham, MA) containing Glutamax-I (L-alanyl-L-glutamine), sodium pyruvate, 4500 mg/ml glucose and pyridoxine supplemented with 10% fetal calf serum (FCS) and 1% nonessential amino acids (all Thermo Fisher Scientific) at 37 °C in a humidified atmosphere containing 10% CO_2_. Where indicated, serum starvation was performed using medium including nonessential amino acids without serum. Asynchronous growing cell cultures were used and all experiments were performed with mycoplasma-free cells. Cell lines were authenticated in 2021 using Multiplex Cell Authentication by Multiplexion (Heidelberg, Germany) as described recently (Castro *et al*. 2013) (Supplementary Fig. S1). The SNP profiles were unique. The purity of cell lines was validated in 2021 using the Multiplex cell Contamination Test by Multiplexion (Heidelberg, Germany) as described recently (Schmitt and Pawlita 2009; Castro *et al*. 2013). No interspecies contamination was detected.

### Colony formation assay

For the measurement of substratum-dependent clonogenic survival, single cells were seeded onto either polystyrene (PS) or fibronectin (FN, 1 mg/cm^2^, BD Biosciences, San Jose, CA) 24 h prior to X-ray irradiation as previously described (Cordes *et al*. 2006). After 8 d, cells were fixed with 80% methanol and stained with Coomassie blue. Colonies with more than 50 cells were counted.

### Radiation exposure

Cells were irradiated at room temperature at different single doses (0–6 Gy) of 240 kV X-rays with a dose rate of approximately 1 Gy/min at 13 mA filtered with 3 mm Beryllium (Isovolt 320/10; Seifert, Ahrensburg, Germany). The absorbed dose was measured using a Duplex dosimeter (PTW, Freiburg, Germany).

### Inhibitor treatment

Inhibition of the EGFR was achieved by BIBX1382BS (20 mM in DMSO, Boehringer Ingelheim, Ingelheim am Rhein, Germany). For inhibition of PI3K, the compound LY294002 (1.25 mM in ethanol, Sigma-Aldrich, Taufkirchen, Germany) was used. Where indicated, cells were serum-starved or grown in complete medium for 16 h in the presence or absence of BIBX1382BS and LY294002 inhibitors prior to irradiation.

### Cell cycle analysis

All cell cultures were grown for 72 h on PS or FN as indicated before cell cycle assessment as previously described (Cordes *et al*. 2006). For steady state cell cycle, cells were analyzed at 0, 4, 8, 12, and 24 h points in time. Radiation-induced cell cycle effects were examined 12 h after irradiation with 6 Gy X-rays. Pharmacological inhibitors were incubated for 16 h prior to X-ray irradiation. In brief, cells were incubated with 1 mM bromodeoxyuridine (BrdU, Serva, Heidelberg, Germany) 10 min prior to harvesting with trypsin/ethylenediaminetetraacetic acid (EDTA) (Thermo Fisher Scientific). Cells were washed with 1 × phosphate-buffered saline (PBS), fixed in 80% ethanol, and subsequently incubated with ribonuclease type III-A (0.01% in 1 × PBS, Sigma-Aldrich), pepsin (0.7 FIP-U, Merck, Darmstadt, Germany), and hydrochloric acid (2 N, Merck). For BrdU staining, mouse anti-BrdU primary antibodies (BD Biosciences) and fluorescein isothiocyanate (FITC) conjugated rabbit anti-mouse secondary antibodies (Agilent Technologies, Santa Clara, California) were used. Total DNA staining was accomplished with propidium iodide solution (Sigma-Aldrich). A FACS Calibur (BD Biosciences) was used for data acquisition. Cell cycle distribution analysis was performed with the CELLQuest software (BD Biosciences).

### Total protein extracts and western blot

Cells were lysed with modified RIPA buffer consisting of 50 mM Tris–HCl (pH 7.4), 1% Nonidet-P40, 0.25% sodium deoxycholate, 150 mM NaCl, 1 mM EDTA, 1 mM NaVO_4_, 2 mM NaF (all Sigma-Aldrich), and complete protease inhibitor cocktail (Roche, Basel, Switzerland). Total protein amount in lysates was quantified by BCA assay (Thermo Fisher Scientific). After SDS–PAGE and transfer of proteins onto nitrocellulose membrane (GE Healthcare, Braunschweig, Germany), probing of specific proteins was accomplished using indicated primary antibodies and horseradish peroxidase (HRP)–conjugated secondary antibodies. ECL Prime western blotting detection reagent (GE Healthcare) was used for the detection of proteins using the Fusion FX imaging device (Vilber Lourmat GmbH, Eberhardzell, Germany).

### Antibodies

Antibodies were purchased as follows: β-actin (A5441, Sigma-Aldrich), β1 integrin (4706, Cell Signaling Technology, Frankfurt am Main, Germany), EGFR (2232, Cell Signaling Technology), αV integrin (4711, Cell Signaling Technology), α5 integrin (98,204, Cell Signaling Technology), α8 integrin (sc365798, Santa Cruz Biotechnology, Dallas, Texas), α9 integrin (NBP2-16,972, Novus Biologicals, Wiesbaden, Germany), Caspase-3 (9662, Cell Signaling Technology), HRP-conjugated sheep anti-mouse secondary antibodies (NXA931, GE Healthcare), and HRP-conjugated donkey anti-rabbit secondary antibodies (NA934V, GE Healthcare).

### Statistical analysis

Data are represented as mean ± SD of two to three independent experiments as indicated. Statistical analysis of the data was performed with Microsoft Excel® using an unpaired *t*-test. *P* values less than 0.05 are considered statistically significant.

## Results

### Role of integrin and growth factor receptor signaling for basal clonogenicity

**Figure 1.**
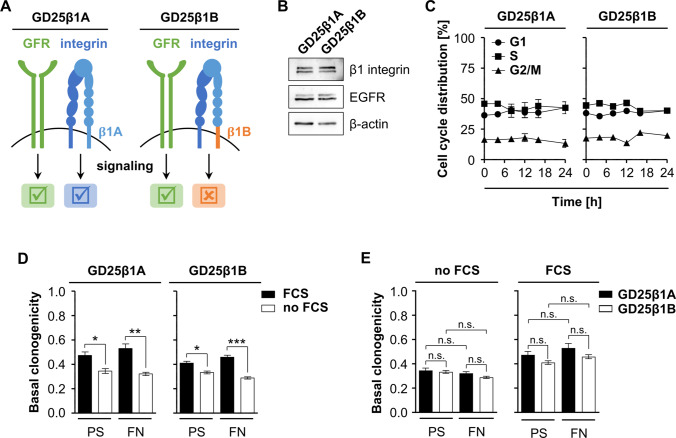
Growth factor receptor signaling facilitates basal clonogenic survival. (*A*) Scheme depicting signaling competences of GD25β1A and GD25β1B cells. (*B*) Representative western blot of basal β1 integrin, EGFR and β-actin expression in GD25β1A and GD25β1B cells. β-actin serves as loading control. (*C*) Basal cell cycle distribution of GD25β1A and GD25β1B cells. Results show mean ± SD (*n* = 3). (*D*, *E*) Basal clonogenic survival of GD25β1A and GD25β1B cells grown on polystyrene (PS) or fibronectin (FN) with or without fetal calf serum (FCS). Results show mean ± SD (*n* = 3; *t*-test; *P < 0.05; **P < 0.01, ***P < 0.001, n.s. non-significant).

To investigate the crosstalk between β1 integrin and growth factor receptor signaling, we employed GD25 β1 integrin knockout fibroblasts, expressing either a fully functional β1A integrin subunit (GD25β1A) or a signaling incompetent β1B integrin splice variant (GD25β1B) (Fig. 1*A*). Western blot analysis shows comparable expression levels of the respective β1 integrin subunit as well as similar levels of the epidermal growth factor receptor (EGFR) in both cell lines (Fig. 1*B*). Furthermore, under steady state cell culture conditions on PS in the presence of FCS, both cell lines demonstrated similar cell cycle distribution profiles over a period of 24 h (Fig. 1*C*). Next, we plated GD25β1A and GD25β1B cells onto either PS or FN in presence or absence of serum to investigate the contribution of β1 integrin signaling and growth factor receptor signaling to basal clonogenic cell survival. In line with the cell cycle profiles showing no difference, both cell lines showed similar levels of survival with significant differences between serum versus starvation conditions (Fig. 1*D*). Furthermore, clonogenic cell survival did not differ between GD25β1A and GD25β1B cells whether they were grown on PS or FN (Fig. 1*E*). These data suggest that basal clonogenic survival is regulated independent from β1 integrin but dependent on growth factor receptor signaling.

### Contribution of integrin and growth factor receptor signaling to radiation survival and radiation-related cell cycle modulations.

**Figure 2.**
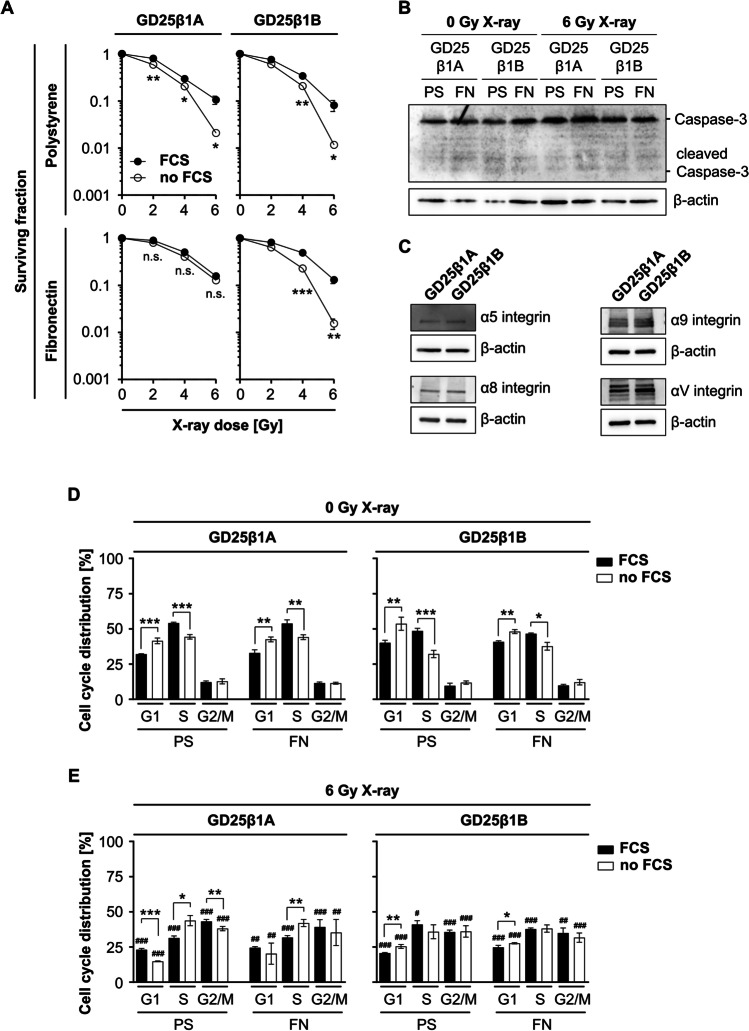
Crosstalk of β1 integrin and growth factor receptor signaling regulates clonogenic radiation survival. (*A*) Clonogenic survival of irradiated GD25β1A and GD25β1B cells on polystyrene (PS) or fibronectin (FN) with or without fetal calf serum (FCS). (*B*) Representative western blot of caspase-3, cleaved caspase-3 and β-actin expression in serum starved GD25β1A and GD25β1B cells on PS or FN before and 24 h after 6 Gy X-ray irradiation. (*C*) Representative western blot of the integrin α4, α5, α8, α9, αV subunits and corresponding β-actin expression in GD25β1A and GD25β1B. (*D*, *E*) Cell cycle distribution of (*D*) unirradiated and (*E*) irradiated GD25β1A and GD25β1B cells on PS or FN with or without FCS. (*A*–*E*) Results show mean ± SD (*n* = 3); *t*-test FCS vs. no FCS; *P < 0.05; **P < 0.01, ***P < 0.001; *t*-test 0 Gy vs. 6 Gy; ^#^*P* < 0.05; ^##^*P* < 0.01, ^###^*P* < 0.001.

As integrins modulate the radiosensitivity of normal and tumor cells, we investigated clonogenic survival of X-ray irradiated GD25β1A and GD25β1B cells grown on PS and FN both in the presence and absence of serum. While serum depletion reduced the clonogenic survival of PS-adherent cells independent from β1 integrin, GD25β1A but not GD25β1B were able to compensate for the lack of serum when plated onto FN (Fig. 2*A*). To examine, whether the reduced clonogenic survival under serum starvation was related to an induction of apoptosis, we analyzed caspase-3 and cleaved caspase-3 expression before and 24 h post 6 Gy X-ray irradiation in both cell lines plated on PS or FN. Western blot analysis showed no differences in caspase-3 expression between both cell lines and treatment conditions (Fig. 2*B*). Furthermore, no cleaved caspase-3 expression was detected, indicating a lack of apoptosis induction (Fig. 2*B*). To investigate, whether differences in the expression of alpha integrin subunits account for the reduced radiation survival of GD25β1B, we assessed the expression of the integrin α4, α5, α8, α9, and αV subunits, which all associate with the integrin β1 subunit to form fibronectin-binding heterodimers. While α4 was not detectable (data not shown), the expression of α5, α8, and α9 did not differ between both cell lines (Fig. 2*C*). Interestingly, we observed an increased αV expression in GD25β1B cells, suggesting a mechanism compensating for the signaling incompetency of the β1B integrin splice variant through other fibronectin receptors such as αVβ3 (Fig. 2*C*). Next, we assessed whether the observed differences in clonogenic radiation survival of both cell lines relate to changes in the cell cycle. To do this, we measured the cell cycle distribution of GD25β1A and GD25β1B cells 12 h post 6 Gy X-ray irradiation compared to unirradiated controls. In line with the data of basal cell survival (Fig. 1*B*), unirradiated GD25β1A as well as GD25β1B cells showed an enrichment of cells in the S phase as response to serum presence independent of whether they were grown on PS or FN (Fig. 2*D*). Furthermore, irradiation significantly increased the percentage of GD25β1A cells in the G2/M phase along with a decrease in G1 and S when compared to unirradiated cells (Fig. 2*E*). This distribution was significantly more pronounced in the presence than the absence of serum on both PS and FN (Fig. 2*E*). In addition, the percentage of GD25β1B cells in the G2/M phase also increased after irradiation on PS and FN; however, this was independent from the presence of serum (Fig. 2*E*). In contrast to basal clonogenicity, these data suggest an important function of β1 integrin for clonogenic radiation survival in addition to growth factor receptors. Thereby β1 integrin signaling seems to cooperate with growth factor receptor signaling to facilitate an enhanced G2/M cell cycle block leading to improved radiation survival.

### β1 integrin and EGFR signaling jointly regulate radiation-induced G2/M cell cycle arrest

**Figure 3.**
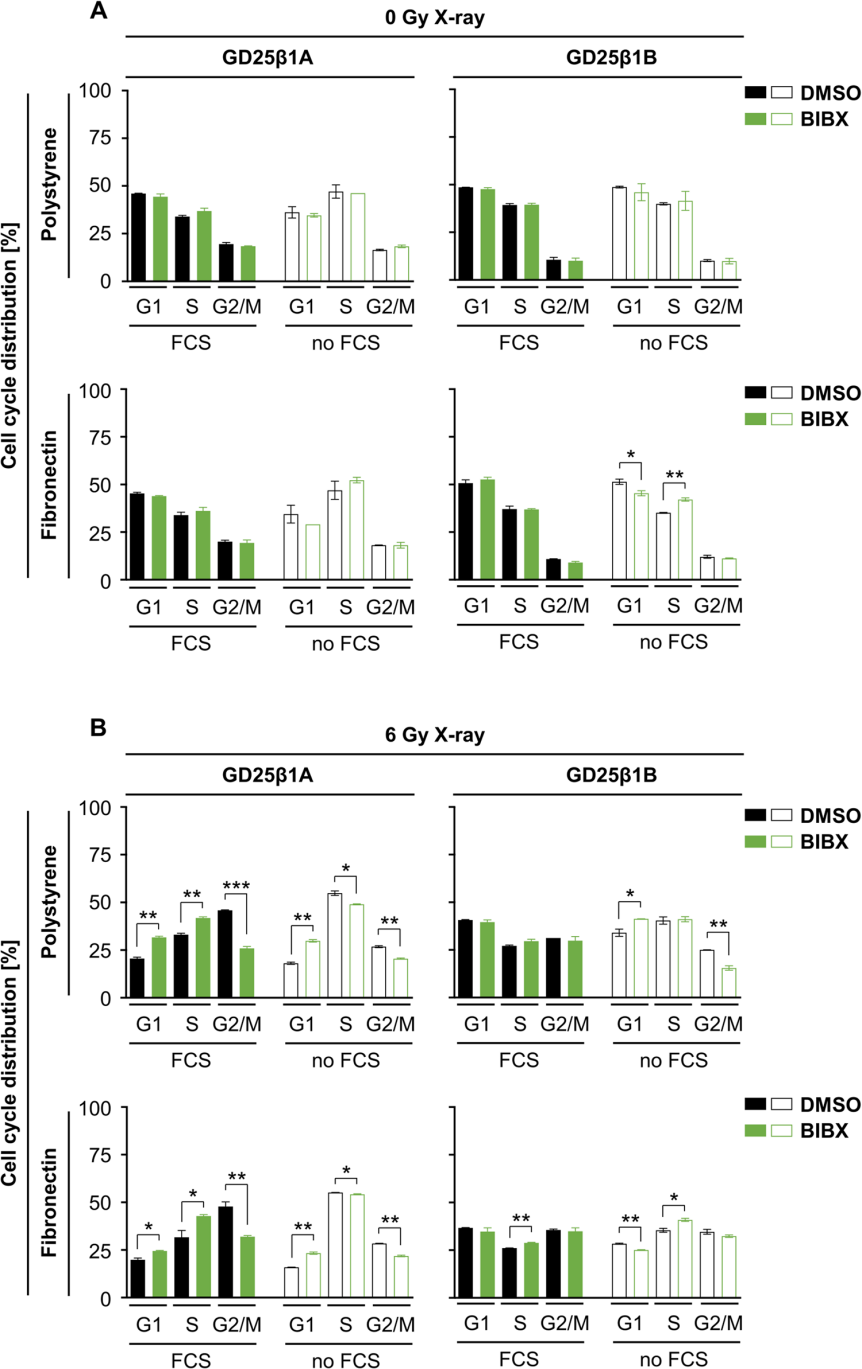
Joint regulation of radiation-induced G2/M cell cycle arrest by β1 integrin and EGFR signaling. (*A*) Cell cycle distribution of unirradiated, DMSO or BIBX1382BS treated GD25β1A and GD25β1B cells grown on polystyrene or fibronectin with or without fetal calf serum (FCS). (*B*) Cell cycle distribution of 6 Gy X-ray irradiated, DMSO or BIBX1382BS treated GD25β1A and GD25β1B cells grown on polystyrene or fibronectin with or without FCS. (*A*, *B*) Results show mean ± SD (*n* = 2); *t*-test; *P < 0.05; **P < 0.01; ***P < 0.001).

To understand the radiation-dependent cell cycle modulation observed in the GD25β1A and GD25β1B cell models better, we chose to inhibit EGFR signaling as known bypass mechanisms to β1 integrin (Eke *et al*. 2015). While administration of the pharmacologic EGFR inhibitor BIBX1382BS alone failed to affect cell cycle distribution of unirradiated GD25β1A or GD25β1B cells (Fig. 3*A*), irradiated cells demonstrated reduced radiation-induced G2/M cell cycle arrest in GD25β1A but not GD25β1B cells independent from serum and FN (Fig. 3*B*). This was paralleled by increased levels of G1 and S phase cell populations (Fig. 3*B*). Jointly, these data highlight the essential contribution of EGFR signaling and its co-dependence on the β1 integrin signaling axis to execute a G2/M cell cycle arrest upon irradiation.

### Radiation-induced G2/M cell cycle arrest codepends on PI3K signaling

**Figure 4.**
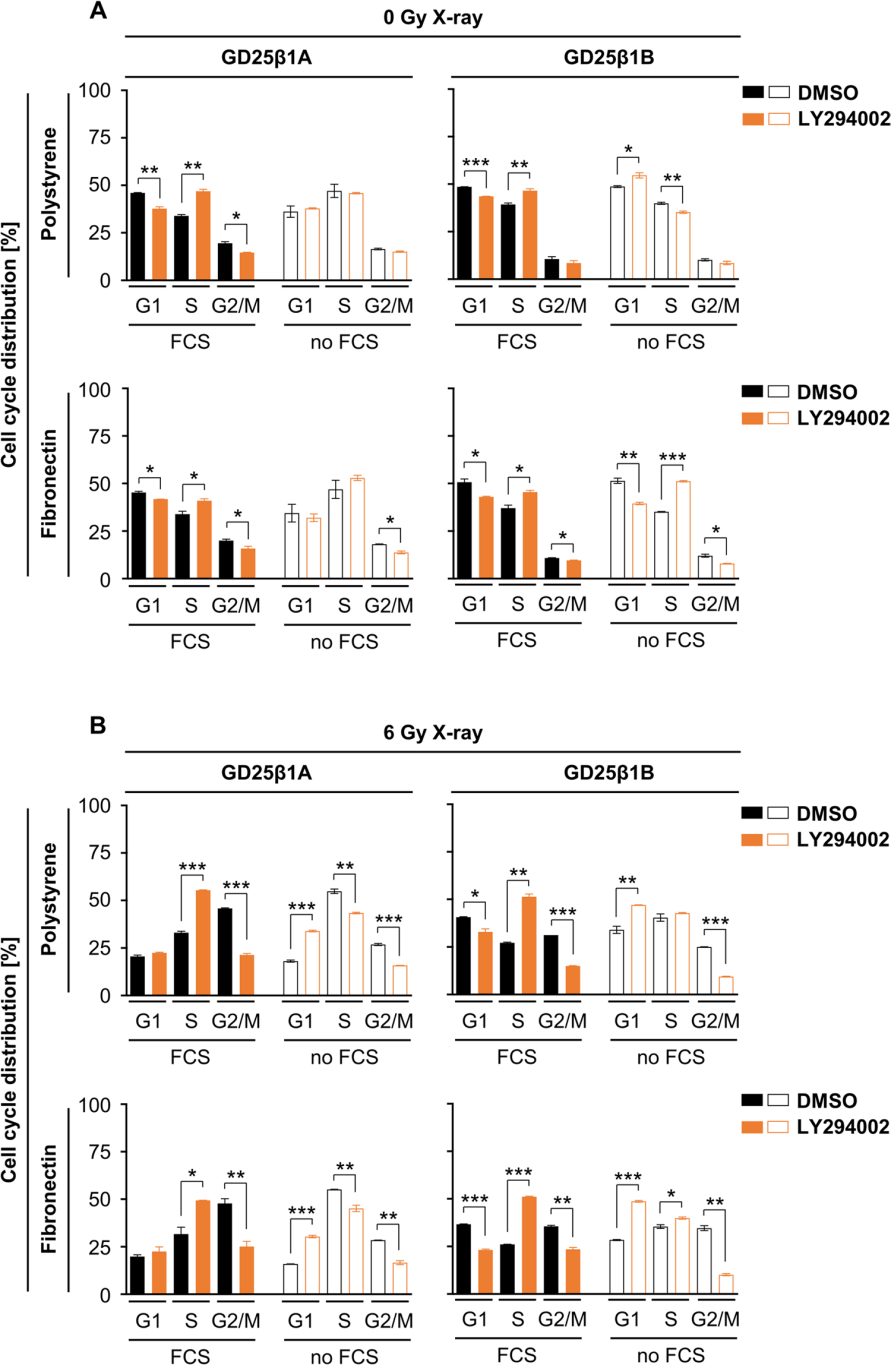
Radiation-induced G2/M cell cycle arrest codepends on PI3K. (*A*) Cell cycle distribution of unirradiated, DMSO or LY294002 treated GD25β1A and GD25β1B cells grown on polystyrene or fibronectin in the absence or presence of fetal calf serum (FCS). (*B*) Cell cycle distribution of 6 Gy X-ray irradiated, DMSO or LY294002 treated GD25β1A and GD25β1B cells grown on polystyrene or fibronectin in the absence or presence of FCS. (*A*, *B*) Results show mean ± SD (*n* = 2); *t*-test; *P < 0.05; **P < 0.01; ***P < 0.001).

To examine whether PI3K as joint signaling determinant downstream of both EGFR and β1 integrin is involved, we pharmacologically deactivated PI3K by LY294002. In line with its prominent pro-survival function and its requirement for G1 cell cycle progression (Vadlakonda et al. 2013), PI3K inhibition declined the percentage of G1 phase cells under basal unirradiated conditions mainly in the presence of serum in both GD25β1A and GD25β1B cell models (Fig. 4*A*). Upon irradiation, however, LY294002 significantly lowered the accumulation of cells in the G2/M phase in both cell models, which was associated with an increased S phase and G1 phase fraction in the presence and absence of serum, respectively (Fig. 4*B*). Intriguingly, these data suggest that the joint regulation of the radiation-induced G2/M cell cycle arrest by the EGFR and β1 integrin pair occurs independent from PI3K. Our observations highlight PI3K to essentially function in cell cycling, especially at the S-G2/M transition in genotoxically injured cells.

## Discussion

Adhesion to the extracellular matrix mediates radio- and chemotherapy resistance through pro-survival integrin signaling. An even better understanding of growth factor receptor/integrin interactions might be beneficial for the advancement of anti-cancer therapies. Here we investigate the crosstalk of β1 integrin and growth factor receptor signaling on clonogenic survival and cell cycling. In the GD25 fibroblast model, we show that (i) competent β1 integrin signaling and growth factor presence seem to be key for clonogenicity upon X-ray irradiation and a pronounced radiogenic G2/M arrest, (ii) the EGFR dependency of the radiation dependent G2/M arrest relies on functional β1 integrin signaling, and (iii) the joint regulation of the G2/M arrest by the EGFR and β1 integrin occurs independent of PI3K.

The formation of a G2/M phase block after ionizing radiation represents a natural reaction of cells to irradiation (Jeggo and Lobrich 2006). Cells mutated in DNA repair proteins, such as ataxia telangiectasia (ATM), do not develop the G2 phase block and are hindered to carry out sufficient DNA repair (Lavin 2008). Compared to GD25β1B cells, GD25β1A cells show a more pronounced G2 phase block after exposure to 6 Gy X-rays when grown in the presence of serum. Hence, the signaling ability of β1 integrin seems essential for the complete development of the postradiogenic G2 phase block. This observation agrees with the results of another study demonstrating an accumulation of human lung fibroblasts in the G2 cell cycle phase when grown on FN along with an increased postradiogenic survival (Cordes and van Beuningen 2004). These data suggest that β1 integrins facilitate the G2/M cell cycle arrest in dependence of growth factor receptor signaling, which allows an optimization of DNA repair mechanisms to facilitate an increased cellular radiation survival.Under EGFR inhibition, serum-grown GD25β1A cells show an increase in the S phase along with a reduction of the G2/M arrest when compared to GD25β1B cells. Thus, S-G2/M transition is hampered and the formation of the postradiogenic G2/M phase block depends not only on EGFR signaling but also on its cooperation with β1 integrin signaling networks. This observation is in line with several reports presenting the synergism between β1 integrin and EGFR signaling pathways (Streuli and Akhtar 2009; Eke *et al*. 2015; Cruz da Silva *et al*. 2019). Therefore, both β1 integrin–mediated signal transduction and an intact EGFR are necessary for an adequate cellular reaction to ionizing radiation.

Inhibition of PI3K, generally known as signaling mediator downstream of both EGFR and β1 integrins (Chong and Janne 2013; Cooper and Giancotti 2019), prevented the formation of the postradiogenic G2/M phase block independent from the signaling ability of β1 integrins. Interestingly, our recent study showed similar effects in human lung fibroblasts grown on PS but not FN, where G2/M blockage was not prevented by PI3K inhibition (Cordes and van Beuningen 2004). These data argue for cell type–specific differences with regard to β1 integrin function in cells exposed to X-rays. Consequently, the functionality of PI3K together with the signaling competence of β1 integrin is essential for a physiological response of fibroblasts to ionizing irradiation.

Mechanistically, integrins and EGFR coalesce together with a plethora of cytoplasmic molecules at focal adhesions. These membrane structures serve to bi-directionally link the extracellular space with intracellular signaling cascades and regulate many normal cellular functions such as cell survival, cell cycle progression, and proliferation, but are also linked to the cellular response to ionizing irradiation (Zaidel-Bar *et al*. 2007; Eke and Cordes 2015). Especially in tumor cells, radiosensitization upon β1 integrin and EGFR inhibition is gained by a reduced repair of radiation-induced DNA double strand breaks (Cordes *et al*. 2003; Kriegs *et al*. 2010; Eke *et al*. 2012; Dickreuter *et al*. 2015). These effects are associated with a deactivation of early downstream signaling events, such as signal transduction through focal adhesion kinase (FAK) as well as a modulation of components the DNA repair machinery (Kriegs *et al*. 2010; Dickreuter *et al*. 2015). As FAK has been suggested to link EGFR and integrin signaling (Eberwein *et al*. 2015), an effect of the signaling deficient β1B variant on DNA repair seems conceivable.

## Conclusions

Taken together, our data suggest β1 integrin as an important determinant of survival and cell cycle distribution of fibroblasts after exposure to ionizing irradiation. This pro-survival effect is possibly due to cooperation with EGFR signaling and independent on PI3K function. Interestingly, these aspects are a remarkable feature of the integrin connection with a variety of growth factor receptors, which facilitate adaptation of cancer cells to therapeutic treatments (Cruz da Silva *et al*. 2019). Further intensified studies are necessary to unravel the underlying molecular circuits and may contribute to the optimization of individualized cancer therapy.

## Supplementary Information

Below is the link to the electronic supplementary material.

Supplementary file1 (TIF 1223 KB)
