# Supplementary figures and images for: Growth factor receptor and β1 integrin signaling differentially regulate basal clonogenicity and radiation survival of fibroblasts via a modulation of cell cycling
Source: In Vitro Cell Dev Biol Anim. 2022 Feb 22;58(2):169–78. doi: 10.1007/s11626-022-00656-z (PMC8901520; doi:10.1007/s11626-022-00656-z)

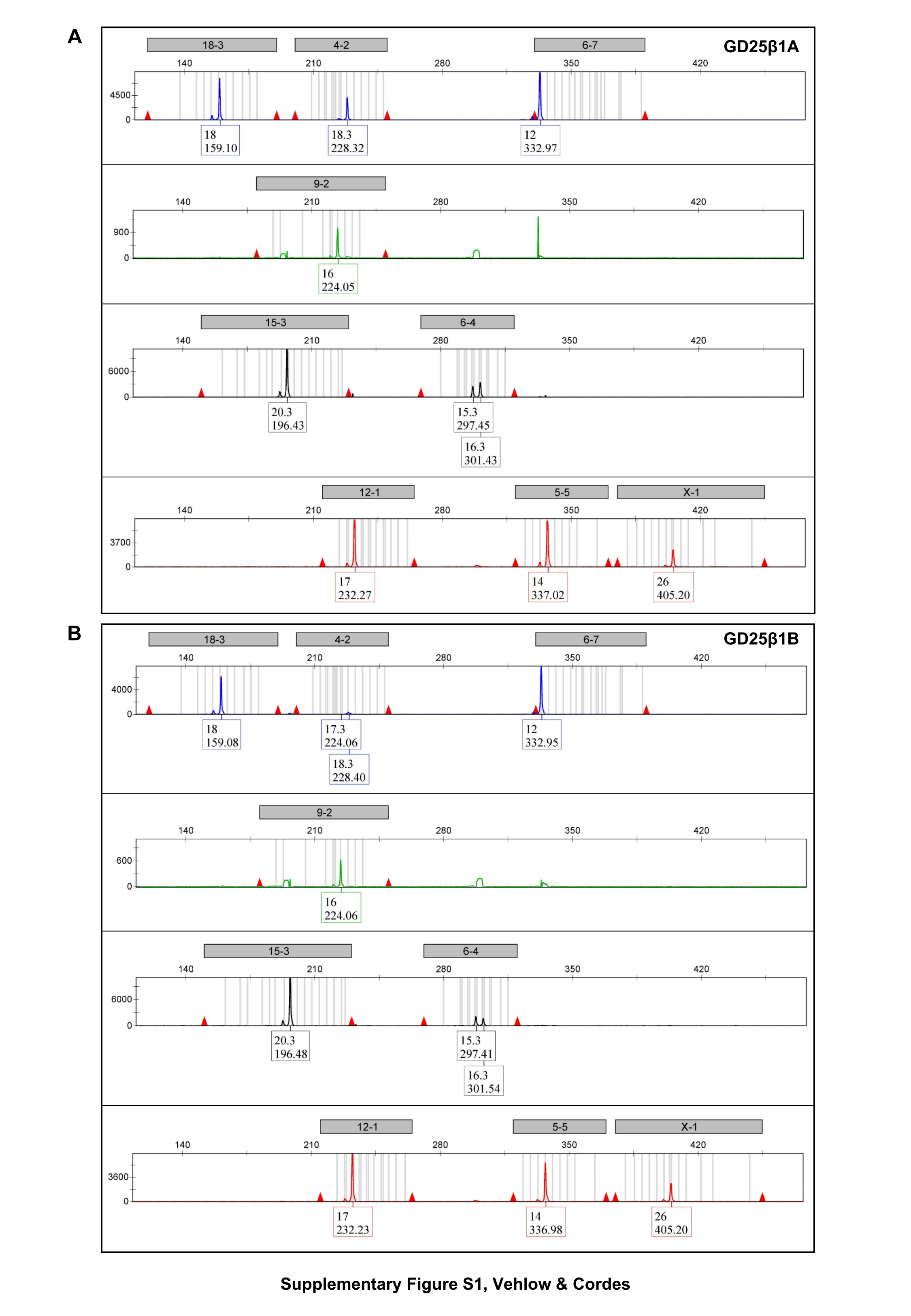

Supplement: Supplementary file 1 — Supplementary file1 (TIF 1223 KB) [file 11626_2022_656_MOESM1_ESM.tif]
